# The Development of a Digital Patient-Reported Outcome Measurement for Adults With Chronic Disease (The Parsley Symptom Index): Prospective Cohort Study

**DOI:** 10.2196/29122

**Published:** 2021-06-11

**Authors:** Hants Williams, Sarah Steinberg, Robin Berzin

**Affiliations:** 1 School of Health Technology and Management Stony Brook University Stony Brook, NY United States; 2 Parsley Health New York, NY United States

**Keywords:** patient-reported outcomes, PROMs, chronic diseases, symptom management, Parsley Symptom Index, Review of Symptoms

## Abstract

**Background:**

The monitoring and management of chronic illness has always been a challenge. Patient-reported outcome measures (PROMs) can be powerful tools for monitoring symptoms and guiding treatment of chronic diseases, but the available PROM tools are either too broad or too disease specific for the needs of a primary care practice focused on longitudinal care.

**Objective:**

In this study we describe the development and preliminary validation of the Parsley Symptom Index (PSI).

**Methods:**

This prospective cohort study took place from January 5, 2018, to June 05, 2020, among a sample of 4621 adult patients at Parsley Health. After a review of literature, followed by binning and winnowing of potential items, a 45-item PROM that also served as a review of systems (ROS) was developed. The PSI was deployed and completed by patients via an online portal. Construct and face validity was performed by clinicians, tested on patients, and feasibility was measured by response rate, completion rate, and percentage of missing data.

**Results:**

The response rate for 12,175 collected PSIs was 93.72% (4331/4621) with a 100% item completion rate. A confirmatory factor analysis confirmed the model structure was satisfactory by a Comparative Fit Index of 0.943, Tucker–Lewis index of 0.938, and root mean square error of approximation of 0.028.

**Conclusions:**

A 45-item ROS-style PROM designed to capture chronic disease symptoms was developed, and preliminary validation suggests that the PSI can be deployed, completed, and helpful to both patients and clinicians.

## Introduction

Chronic disease is now the primary cause of death and disability in the United States [1] and accounts for 90% of the nation’s US $3.5 trillion in annual health care costs [2]. The incidence of chronic disease is on the rise, and people are developing chronic diseases long before they are bedbound, hospitalized, or even symptomatic. Epidemiological data indicate that chronic diseases such as diabetes, heart disease, and asthma are underdiagnosed up to 90% of the time in the developed world [3]. Current models also predict that prevalence of chronic disease will reach 80% by 2030 [2]. The existing health care system was designed for acute illness and is poorly suited for chronic disease, which now accounts for the majority of services provided [4]. The massive increase in chronic disease is rapidly unmasking the gaps in our health care delivery system, particularly in disease monitoring and management.

The monitoring and management of chronic disease is a challenge regardless of a medical practice size or physical location. For clinicians, a limited number of chronic disease monitoring and management tools exist that can be quickly deployed into the patient–clinician workflow, can integrate with an electronic medical record system, can be utilized across a variety of different conditions, can supplement a review of systems (ROS), are clinically validated, and can be brief enough to be collected on an ongoing basis. Patient-reported outcome measures (PROMs) can be incredibly helpful for monitoring and guiding treatment of chronic diseases [5-7]. Examples of PROMs that have been created to address some of the aforesaid challenges range from single-form assessments such as the Medical Symptom Questionnaire (MSQ) [8], which are akin to a ROS, to the Patient-Reported Outcomes Measurement Inventory System (PROMIS) that offers variations for general health and specific conditions [9].

The MSQ assesses physical symptoms in a brief form, and serves as a ROS within a medical note. However, the MSQ has yet to be validated and has inconsistent categorization of items. The items included within the MSQ range from clinical diagnoses such as asthma and arthritis, vital sign measurements such as weight, and symptoms that range from slurred speech to drainage from the ear. The MSQ also has complex instructions where the user must assess each item’s frequency and severity at the same time, regardless of whether the item is for a symptom, condition, or vital sign. These and other concerns make the MSQ difficult to integrate into a clinician’s workflow and patient visits.

By contrast, the PROMIS is one of the most rigorously developed sets of PROMs, and covers a wide range of chronic diseases in both short- and long-form versions [10] that capture physical and psychosocial domains. In comparison to the MSQ, the PROMIS short forms focus heavily on psychological well-being and overall quality of life questions, but not extensively enough to double as a ROS like the MSQ. PROMIS has also developed specific variations for individual chronic diseases [11]. The technical deployment of PROMIS variations (short versus long form, condition specific) can be managed by the information technology departments that exist within large medical practices (eg, academic medical centers), but can be burdensome for a small primary care practice without those resources.

Both the PROMIS and the MSQ are powerful tools in their own right, but neither offer a single, short-form assessment that could be easily integrated into the clinician workflow or electronic medical record, as well as capture symptoms across body systems like a ROS. Furthermore, neither allows for more opportunities to engage with patients like a digital health tracker (eg, smart watch, fitness tracker). Approximately 1 in 5 US adults say they regularly wear a digital tracker that can collect health information [12]. While the data collected from these devices can be motivational and promote behavioral change [13], long-term engagement is still a challenge for these trackers [14]. In summary, we envision a new type of PROM that could function like a ROS, feed into the collaborative patient–doctor conversation to promote personalized tailoring of care plans, while also offering opportunities for more continuous engagement like a digital health tracker. The aim of this study is to (1) describe the initial development of the Parsley Symptom Index (PSI) and (2) assess feasibility of the PSI among patients receiving care at Parsley Health.

## Methods

### Setting and Population

Parsley Health is a subscription-based model for delivering primary care and proactive chronic disease management through a functional medicine lens. Patients receive care from clinicians and health coaches in-person and virtually, and have additional access to their care team via email and a web portal. Parsley Health patients are predominantly female (85%), range from 18 to 83 years in age (mean age 37 [SD 6.7]), and are located primarily in metropolitan areas such as New York City and Los Angeles. Commonly reported diseases and health problems (ICD-10 chapters) for patients seeking treatment include mental, behavioral, and neurodevelopmental disorders (F41.9, Anxiety disorder; R53.83, Fatigue, G47, Insomnia), digestive system (K21.9, Gastro-esophageal Reflux Disease; K90.41, Non-celiac Gluten Sensitivity; K58.0, Irritable Bowel Syndrome), and diseases of the skin and subcutaneous tissue (L70.9, Acne; L20.9, Atopic Dermatitis).

Inclusion criteria were Parsley Health patients that had (1) an active “12-month complete care” membership between January 12, 2018, and June 05, 2020; (2) a minimum of 1 clinical visit within their membership period; and (3) located at 1 of the 3 locations: New York City, Los Angeles, or San Francisco. Exclusion criteria were (1) severe psychiatric disorders (particularly psychosis and depression requiring a change in treatment in the last 30 days); (2) under the age of 18; (3) unable to speak or read English; or (4) lacked access to a computer.

### PSI Development

#### Overview

The PSI development and testing followed the frameworks outlined by the Federal Drug Agency (FDA) guide for PROM development [15], and the PROMIS investigators [9,16]. Our approach included initial item identification by literature review and secondary data analysis of 2 US national health surveys, item classification and selection (binning and winnowing), focus group exploration followed by item revision, and field testing followed by further revision. This study involved patient-reported survey data that were recorded in such a manner that participants could not be identified by the researchers. The institutional review board at Stony Brook University considered this study exempt from 45 Code of Federal Regulations requirements [17].

#### Review of Literature and Secondary Data Analysis

Item development started with a systematic literature review of PROM for adult patient populations. Our initial PubMed search was performed using the MeSH terms for PROMs: “patient outcome assessment” and “patient reported outcome measures.” This search returned a list of 342 systematic literature review articles. The vast majority of results returned by the search focused on individual chronic diseases and quality of life (QoL). Subsequent searches were focused on the identification of the most prevalent chronic diseases and their symptoms in the United States defined by the National Health Interview Survey (NHIS) [18] and the National Ambulatory Medical Care Survey (NAMCS) [19].

A secondary analysis of data pulled from the 2015 NHIS survey included a subsample of 17,201 unique patient responses between the ages of 18 and 50, and a sample of 3583 physician responses and 76,330 completed patient record forms from the 2012 NAMCS. Chronic diseases and their symptoms described in the NHIS and NAMCS were combined with the initial PROM MeSH terms to create additional PubMed searches. Approximately 320 individual items from 27 PROMs were inspected and entered into a document by our clinical team members to ensure the item list reflected common clinical symptoms.

#### Binning and Winnowing

**Figure 1:**
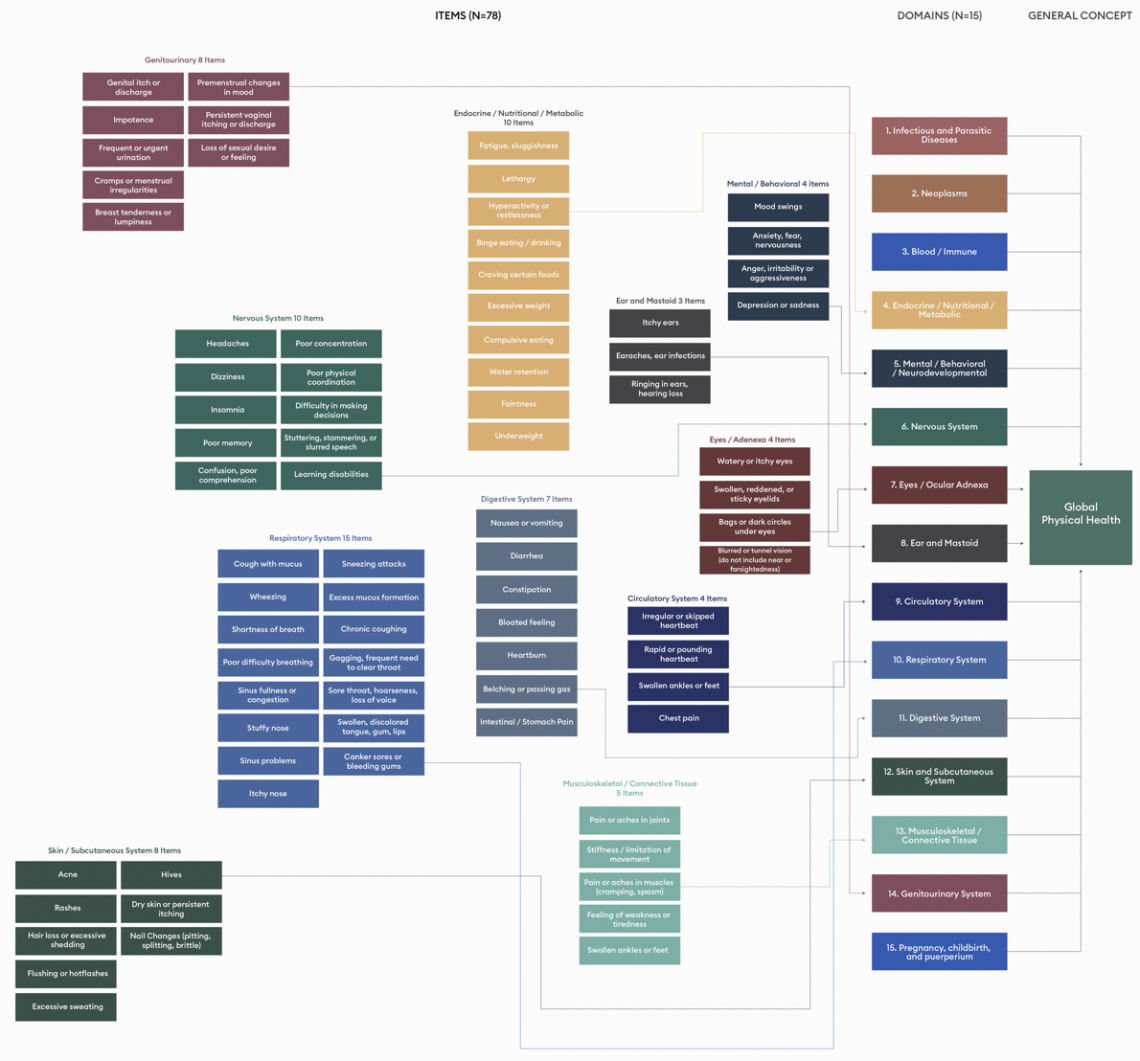
Original PSI design: 78 items sorted into 15 domain bins. PSI: Parsley Symptom Index.

Binning is a systematic process that groups items by meaning and construct, while winnowing is used to reduce an item pool to a representative and manageable set of items [16]. The clinician study team independently sorted 320 items into bins according to meaning and construct in order to identify redundant items, and ensure a representative sample of items. At the end of the binning process each team member compared bins and discussed discrepancies. For unresolved discrepancies, an additional team member was brought into the discussion until a resolution was reached. What remained was 78 items sorted into 15 domain bins (Figure 1).

**Figure 2:**
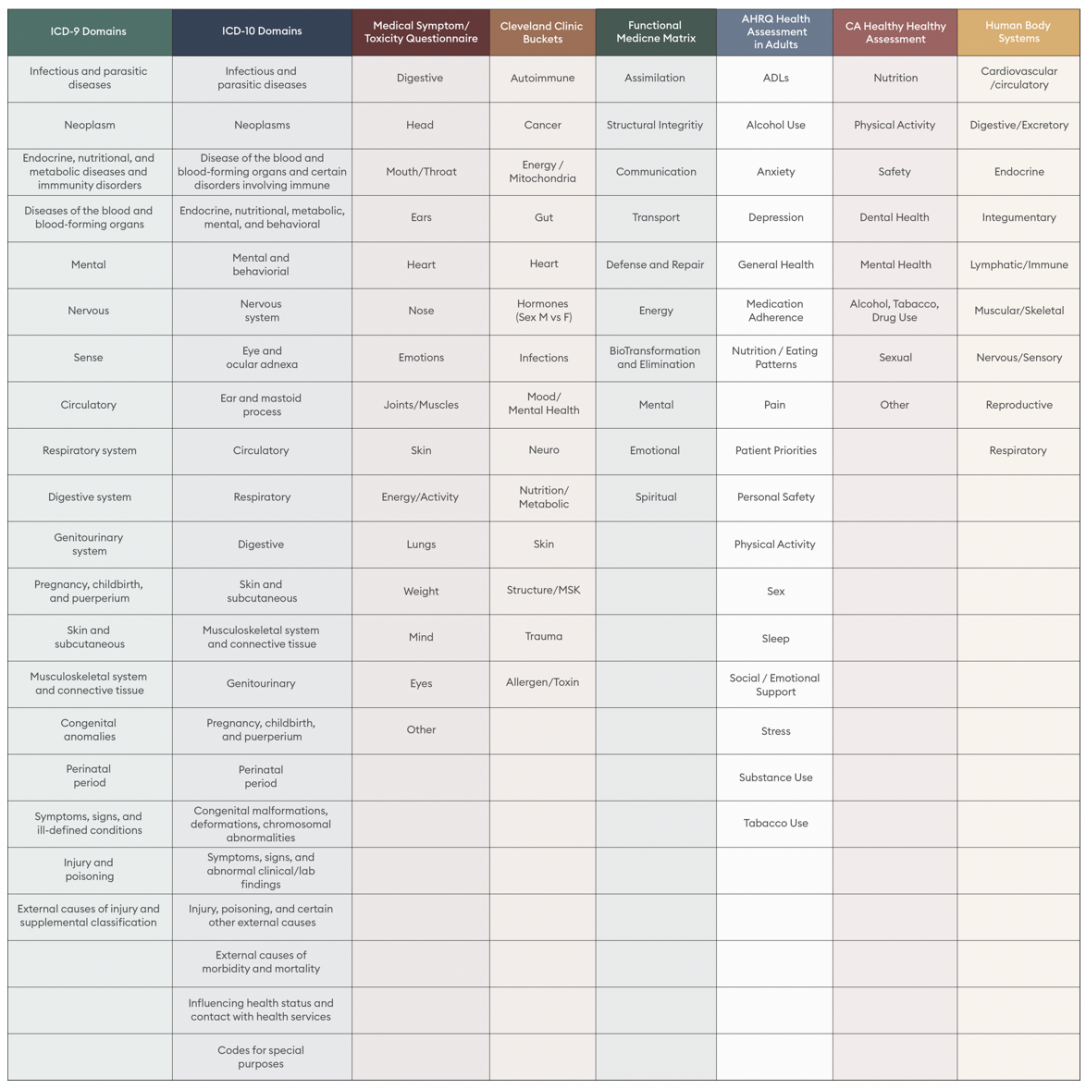
Logic for PSI bin relabeling with patient facing terminology to reflect bodily systems. ADL: activities of daily living; PSI: Parsley Symptom Index.

Next the team winnowed the remaining items and established exclusion criteria for each item in a bin. Exclusion criteria were constructed based on the clinical expertise of the team, and findings from the NHIS and NAMCS reports. A 3-reviewer consensus was required for item removal from a bin. The final result was a set of 45 items sorted into 9 bins (Multimedia Appendix 1). Based on a review of other similar assessments (Figure 2), each bin was relabeled with patient facing terminology to reflect bodily systems: Cardiac and Circulatory, Gastrointestinal, Metabolic, Hair and Skin, Neurological, Respiratory, Musculoskeletal, Mental, and Reproductive (Female and Male). The stem of each item was standardized to ask if the symptom was present or not present; then, double-barreled items (ie, items that assess more than 1 concept) were removed and the response time frame was set to a 14-day period. A 14-day window was selected to minimize recall bias [20] and allow for repeat testing. For questions answered present, an additional exploratory question was displayed to capture the symptom’s intensity on a sliding Visual Analog Scale (VAS) of 1-10. Emoticons (smiley and sad faces) were displayed on each endpoint to clarify the meaning of the scale. A VAS was selected to quantify symptom severity because it is simple to use, requires no training, and is both accurate and sensitive [21]. To allow for straightforward calculation and interpretation by patients and clinicians, a total score was constructed by a sum of the VAS scores for all “yes” answers, with “no” being assigned a value of 0.

To quickly identify gaps in item coverage, time to complete the PSI, and assess clarity of item language, a convenience sample of 76 patients (mean age 34 [SD 4.3]; 62/76, 82% female) provided preliminary feedback [22,23]. Patients were asked to provide open-ended qualitative feedback in a free-text box at the end of the assessment. For 82% (62/76) of patients the PSI took less than 5 minutes to complete, for 14% (11/76) between 5 and 10 minutes, and more than 10 minutes for 4% (3/76). Two team members (HW and RB) reviewed all patient responses to identify missing content or functionality. Patient feedback on individual PSI items were generally positive. Patient feedback on functionality focused on user interface and user experience; examples included the addition of a progress bar, preferences on multipage versus single-page layouts, and how to make the PSI more engaging through the addition of animations and visual changes as the patient completes the PSI. Despite the overwhelming positive feedback, a dilemma did occur for patients that experienced symptoms over the 14-day window that had resolved, which was why a sub category was added to the yes response: “Yes—ongoing” and “Yes—resolved.”

### Integration Into Clinical Workflow

Patients were provided instructions to login to their “My Parsley” web portal to complete the PSI 24-48 hours before each clinical visit. Because the initial visit requires multiple data collection forms (medical, family, and social histories) as well as the PSI, there was greater motivation to complete all the forms for the first visit than for follow-up visits. Initial visits were rescheduled if all the forms were not completed, whereas follow-up visits were never postponed for lack of a completed PSI. To encourage compliance, a clinical operations coordinator looked for a recorded online PSI response before each follow-up visit, and prompted the patient to fill one out if missing.

Patients and providers both interacted with the PSI at several points. Before the clinical visit patients completed the PSI and viewed their score online, which provided them with immediate feedback. Within the clinical visit the PSI score was used as a touch point for the patient–provider discussion, and assisted the clinician by removing the need to spend visit time collecting ROS information. In subsequent visits, clinicians were able to share PSI trend data with patients to support longitudinal care.

### Statistical Approach

Analyses were conducted with Python (version 3.6.4) and R (version 4.0.4) [24]. Descriptive statistics to summarize age, gender, membership duration, and participant location were generated using the Python package TableOne (version 0.7.10) [25]. Feasibility of the PSI was examined by response rate, completion rate, and percentage of missing data. We considered a response rate of over 85% to be adequate [26,27]. The response rate was calculated by determining the percentage of patients who had at least one clinical visit between January 12, 2018, and June 05, 2020, that completed at least one PSI. Skewness, kurtosis, and response distributions were reviewed for each PSI item to help assess relevance and response frequency. To translate the PSI total score into clinically meaningful values, preliminary cut-off points based on quartile ranges were calculated. Lastly, we did not expect missing values due to the fact that only a fully completed PSI can be submitted, so if missing values were to occur, they would be likely related to a software defect.

A confirmatory factor analysis (CFA) was conducted to examine the proposed factor structure of the PSI as opposed to an exploratory factor analysis (EFA). In an EFA each item would be free to load on to any factor, potentially leading to a model that is inconsistent with the theory-derived determination of PSI items and factors, whereas a CFA allows data to be fitted to a theory-derived model, with each item only loading to the factors it was designed to measure, helping to address potential weaknesses of specific items. The minimum sample size for the CFA was calculated with an item-to-respondent ratio of 30:1 [28,29]. Kline [28] notes the N:q rule, which states that the sample size should be determined by the number of q parameters in your model with a recommended ratio that can range from 15:1 to 30:1. With a 45-item assessment, the minimum sample size would be 900 participants based on a 30:1 ratio.

To prepare the data for the CFA, each item was recoded into a numerical dummy variable (0 for symptom not present; 1 for symptom present or resolved). The CFA was conducted in R with the lavaan latent variable analysis package version 0.6.8 [30] using diagonally weighted least squares (DWLS). The DWLS estimator has growing consensus among researchers as the best approach for the analysis of binary variables [31]. Model appropriateness was assessed via the root mean square error of approximation (RMSEA; 0.05 < cut-off < 0.08) [32,33], the Tucker–Lewis index (TLI; cut-off ≥ 0.90) [34], and the Comparative Fit Index (CFI; cut-off ≥ 0.90) [35]. The model was improved based on the removal of items with small factor loadings and the through assessment of expected versus observed counts for each categorical indicator variable. Standardized factor loadings (β) less than 0.30 or CIs below 95% were deemed poorly performing items [36,37]. These cut points were used as a guide rather than strict rules [38]. We focused on the relative size of these indicators to inform choices around item retention and removal, in conjunction with the impact to the overall model fit following item removal, and the theoretical coverage of the remaining items.

## Results

A total of 12,175 PSI-unique assessments were collected from 4621 patients. Females accounted for 80.22% (3707/4621), the mean age was 38.9 years (SD 11), and each patient had an average of 2.96 (SD 1.7) clinician visits per 12-month membership period (Multimedia Appendix 2). The PSI response rate was 93.72% (4331/4621). Over the duration of the study the PSI was completed 1 time by 24.41% (1128/4621), 2-3 times by 43.26% (1999/4621), and 4 or more times by 26.73% (1235/4621) of study patients.

The 3 bodily domains with the highest frequency of present or resolved symptoms were neurological (10,113/12,175, 83.06%), mental (9667/12,175, 79.40%), and gastrointestinal (9428/12,175, 77.44%), while the bodily domain with the lowest frequency was sexual health (3165/12,175, 25.99%). The top reported individual symptoms across all bodily domains were “fatigue or low energy” (7954/12,175, 65.33%), “nervousness or anxiety” (7449/12,175, 61.18%), and “bloating or abdominal pain” (7086/12,175, 58.20%). The normality, skewness, and kurtosis for the reported VAS of each item are displayed in Multimedia Appendix 1.

Quartiles calculated for the total score resulted in the following 4 cut-off ranges: 0-24, 25-43, 44-71, and greater than 71. The clinical study team assigned the following terminology for these ranges: “well” (0-24), “symptomatic” (25-43), “very symptomatic” (44-71), and “sick” (71+). These ranges provide a preliminary rubric that allowed clinicians to quickly interpret the total score and assess changes to symptoms over time.

Of note, the PSI cannot be submitted with incomplete responses. Nonresponders (290/4621, 6.27%) included those who filled out the PSI partially and those who did not fill it out at all. While completion rates for the PSI decreased over time with subsequent clinical visits, clinicians reported that when patients did complete follow-up PSIs, they were helpful for longitudinal tracking and improved their ability to trend symptoms over time. Furthermore, clinicians reported that modeling the PSI on a ROS increased perceived workflow efficiency, and motivated them to encourage their patients to complete the PSI prior to each visit. Additional feedback indicated that the PSI assisted in making the patient feel heard, and provided meaningful context for the visit.

For the CFA, 2 items were initially removed (“hives” from the skin factor and “genital itch” from the male factor) due to having only a single level (symptom not present). The initial fit statistics for the model were satisfactory (CFI=0.929, TLI=0.923, and RMSEA=0.031). Nearly all items had β values >.3 except for “snoring” (β*=*.246). With the removal of “snoring” the model marginally improved (CFI=0.931, TLI=0.925, and RMSEA=0.031). To explore ways of improving the model further, items with high modification indices were investigated for cross-loading. Two items with poor conceptual specificity that loaded onto several other dimensions were “wheezing/chest tightness” within the respiratory factor (high cross-loading onto “shortness of breath” [cardiac] and “chest pain” [cardiac]) and leg swelling from the cardiac factor (high cross-loading onto “joint swelling” [musculoskeletal] and “limited range of motion or function” [musculoskeletal]). The removal of these 2 items improved the model to a small degree (CFI=0.943, TLI=0.938, and RMSEA=0.028).

## Discussion

### Principal Findings

Our goal in designing the PSI was to create a new type of PROM that could function like a ROS, feed into the collaborative patient–doctor conversation to promote personalized tailoring of care plans, and offer opportunities for continuous engagement like a digital health tracker. The preliminary data described within this study set the groundwork for future research that can further assess the efficacy and ecological validity of the PSI, and explore the PSI’s potential impact on the patient–clinician interaction within a visit.

During the data-collection phase of the study, we quickly realized that in asking patients to fill out the PSI, we were competing with digital health trackers for our patients’ engagement. In general, patients have become increasingly interested in tracking their own habits and symptoms [39], and there is an ever-growing patient demand for more engaging monitoring technologies [40,41]. Many of our own patients already use digital health trackers to monitor activities related to physical movement, sleep behaviors, heart rate and blood pressure, weight, and nutritional intake. Most of these trackers involve the recording of one’s own data and receiving immediate feedback, yet the majority still lack the ability to provide feedback to the patient that is personalized and actionable.

In competing for our patients’ engagement, we drew inspiration and borrowed what has worked for digital health trackers and attempted to address their shortcomings. First, we focused on the importance of a user-centered design that emphasizes the importance of the user interface and user experience. In the design process we paid particular attention to the visual styles, design elements, and the overall user experience. Second, because providing immediate results in digital health trackers has been shown to influence behavioral change [42,43], we designed the PSI to provide an immediate result upon completion, a total score. At the current stage in PSI development there is no automated interpretation of the total score. The score is interpreted by the clinician within the patient visit where it is contextualized based on recent patient illnesses, stressors, and treatments. Third, we have addressed the inability of most trackers to provide individualized interpretation of health data by incorporating the results into the clinical encounter. Through this approach the PSI goes beyond generic recommendations, and assists the clinician with creating a personalized, actionable treatment plan that is patient centered. Going forward, we suggest that future enhancements to the PSI and other PROMs are performed through the lens of digital health trackers to expand engagement and utilization beyond a traditional questionnaire.

An important finding within this study was the PSI’s high completion rate (4331/4621, 93.72%) for initial visits. While preliminary data showed PSI completion rates declined after initial visits, clinician feedback was extremely positive for patients that did continue to fill out the PSI for follow-up visits. This further highlights that the monitoring of chronic disease symptoms over a long period is a difficult challenge, regardless of patient condition or technology used.

In our attempt to enhance long-term engagement and completion of the PSI, we have initiated and continue to engage in quality improvement efforts. Two areas of improvement that have been identified by our patients and staff are reminder notifications and reporting of results. Related to notifications, we are exploring the impact of (1) delivery time (day, weekend, morning, evening), (2) phrasing of messaging in reminder notifications, and (3) delivery medium (email, text, telephone). As for PSI reporting, we are exploring (1) addition of longitudinal line graph of total scores, and (2) a stacked line graph visualizing each body system over time. Graphical presentations of data are used to make information “stickier” with existing digital health trackers, so we believe both clinicians and patients may derive further benefits from seeing a picture of their progress over time at the macro level (total score) and micro level (bodily system).

### Limitations

The PSI generalizability may be limited due to our sample being largely female. In future validation studies, testing in populations with greater gender diversity will help improve ecological validity. Second, due to workflow issues around initial data entry, we do not have sufficient data to describe race and ethnicity. Future external validation of the PSI should include testing in diverse patient populations.

### Conclusion

This study details the process and methodology for how the PSI was created. With a response rate of nearly 94% (4331/4621, 93.72%), the initial findings suggest that the PSI can be used in clinical practice. Drawing lessons from digital health trackers, the PSI offers immediate feedback that informs the patient–clinician dialogue, and may promote enhanced tracking and management of chronic disease symptoms over time.

## Appendix

Multimedia Appendix 1 Descriptive statistics of individual PSI items.

Multimedia Appendix 2 Descriptive statistics of sample.

## Abbreviations

PROM: patient-reported outcome measure
PROMIS: Patient-Reported Outcomes Measurement Inventory System
PSI: Parsley Symptom Index
ROS: review of symptoms
TLI: Tucker–Lewis index
