# Supplementary material for: The Development of a Digital Patient-Reported Outcome Measurement for Adults With Chronic Disease (The Parsley Symptom Index): Prospective Cohort Study
Source: JMIR Form Res. 2021 Jun 11;5(6):e29122. doi: 10.2196/29122 (PMC8235288; doi:10.2196/29122)
Supplement: Multimedia Appendix 1 [file formative_v5i6e29122_app2.docx]

Table 2: PSI Item Responses

|  | **Min** | **Max** | **Response Frequency (% (n))** | **Mean Value** | **SD** | **Skewness** | **Kurtosis** |
| --- | --- | --- | --- | --- | --- | --- | --- |
| **Cardiovascular** |  |  |  |  |  |  |  |
| Breath shortness | 1.0 | 10.0 | 9.61% (1168) | 4.97 | 1.92 | 0.24 | -0.34 |
| Chest pain | 1.0 | 10.0 | 8.38% (1018) | 4.81 | 1.89 | 0.32 | -0.25 |
| Fainting | 1.0 | 10.0 | 20.03% (2433) | 4.87 | 1.93 | 0.22 | -0.39 |
| Leg Swelling | 1.0 | 10.0 | 6.94% (843) | 5.03 | 2.0 | 0.09 | -0.57 |
| Rapid or pounding heartbeat | 1.0 | 10.0 | 15.15% (1840) | 5.04 | 1.91 | 0.1 | -0.25 |
| **Female Specific** |  |  |  |  |  |  |  |
| Genital Discharge | 1.0 | 10.0 | 5.54% (673) | 4.02 | 1.99 | 0.48 | -0.26 |
| Genital Itch | 1.0 | 10.0 | 3.51% (427) | 4.58 | 2.07 | 0.46 | -0.42 |
| Irregular or Heavy Periods | 1.0 | 10.0 | 8.87% (1078) | 5.89 | 2.44 | -0.02 | -0.74 |
| Painful Sex | 1.0 | 10.0 | 3.06% (372) | 5.42 | 2.12 | 0.1 | -0.35 |
| Vaginal Dryness or Hot Flashes or Night Sweats | 1.0 | 10.0 | 7.48% (909) | 5.14 | 2.08 | 0.2 | -0.49 |
| **Gastrointestinal** |  |  |  |  |  |  |  |
| Bloating or Abdominal Pain | 1.0 | 10.0 | 58.33% (7086) | 5.74 | 2.05 | 0.08 | -0.52 |
| Diarrhea or Constipation | 1.0 | 10.0 | 49.95% (6068) | 5.57 | 2.05 | 0.13 | -0.47 |
| Excessive Gas | 1.0 | 10.0 | 39.79% (4834) | 5.5 | 2.06 | 0.16 | -0.49 |
| Heartburn or Reflux | 1.0 | 10.0 | 24.41% (2965) | 5.15 | 1.99 | 0.25 | -0.37 |
| Nausea | 1.0 | 10.0 | 20.16% (2449) | 5.42 | 2.04 | 0.2 | -0.48 |
| **Hair and Skin** |  |  |  |  |  |  |  |
| Abnormal Hair Changes | 1.0 | 10.0 | 11.33% (1377) | 5.67 | 2.13 | 0.05 | -0.56 |
| Abnormal Nail Changes | 1.0 | 10.0 | 5.33% (648) | 4.95 | 2.04 | 0.23 | -0.45 |
| Acne | 1.0 | 10.0 | 43.87% (5330) | 4.73 | 2.12 | 0.34 | -0.47 |
| Rashes | 1.0 | 10.0 | 24.22% (2943) | 5.22 | 2.22 | 0.21 | -0.67 |
| **Male Specific** |  |  |  |  |  |  |  |
| Erectile Dysfunction | 2.0 | 10.0 | 0.75% (91) | 6.48 | 2.2 | -0.2 | -0.61 |
| Genital Itch or Discharge | 1.0 | 10.0 | 5.54% (673) | 4.02 | 1.99 | 0.48 | -0.26 |
| Interrupted Urinary Stream | 1.0 | 9.0 | 0.4% (48) | 4.6 | 2.32 | 0.68 | -0.53 |
| Low or Change to Sexual Drive | 1.0 | 10.0 | 1.53% (186) | 5.8 | 2.18 | 0.03 | -0.55 |
| Testicular Lumps or Pain | 1.0 | 9.0 | 0.3% (36) | 4.5 | 2.26 | 0.42 | -0.71 |
| **Mental Health** |  |  |  |  |  |  |  |
| Binge or Restrictive Eating | 1.0 | 10.0 | 14.41% (1751) | 5.25 | 2.14 | 0.17 | -0.55 |
| Depression | 1.0 | 10.0 | 27.66% (3361) | 5.22 | 2.06 | 0.16 | -0.56 |
| Easily Distracted or Feelings of ‘Brain Fog’ | 1.0 | 10.0 | 50.4% (6123) | 5.42 | 2.11 | 0.18 | -0.59 |
| Mood Swings | 1.0 | 10.0 | 42.47% (5160) | 5.22 | 1.98 | 0.14 | -0.51 |
| Nervousness or Anxiety | 1.0 | 10.0 | 61.30% (7449) | 5.42 | 2.09 | 0.13 | -0.66 |
| **Metabolism** |  |  |  |  |  |  |  |
| Abnormal Weight Gain or Loss | 1.0 | 10.0 | 12.54% (1524) | 5.51 | 2.18 | 0.08 | -0.47 |
| Excessive Hunger or Thirst | 1.0 | 10.0 | 13.33% (1620) | 5.42 | 1.93 | 0.08 | -0.39 |
| Frequent Urination | 1.0 | 10.0 | 16.47% (2001) | 5.29 | 2.05 | 0.17 | -0.44 |
| Intolerance to Heat or Cold | 1.0 | 10.0 | 17.82% (2165) | 5.65 | 1.96 | -0.06 | -0.37 |
| Negative Relationship with Food | 1.0 | 10.0 | 16.79% (2040) | 5.37 | 2.1 | 0.24 | -0.54 |
| Vision Changes | 1.0 | 10.0 | 6.12% (743) | 4.88 | 2.02 | 0.3 | -0.31 |
| **Musculoskeletal** |  |  |  |  |  |  |  |
| Backpain | 1.0 | 10.0 | 39.48% (4797) | 5.18 | 2.02 | 0.23 | -0.53 |
| Joint Pain | 1.0 | 10.0 | 40.21% (4885) | 5.1 | 2.04 | 0.24 | -0.57 |
| Joint Swelling | 1.0 | 10.0 | 9.11% (1107) | 5.2 | 2.1 | 0.2 | -0.59 |
| Limited Range of Motion or Function | 1.0 | 10.0 | 17.76% (2158) | 5.51 | 2.09 | 0.05 | -0.56 |
| Muscle Soreness or Cramps | 1.0 | 10.0 | 37.15% (4513) | 4.97 | 1.95 | 0.3 | -0.37 |
| **Neurological** |  |  |  |  |  |  |  |
| Fatigue or Low Energy | 1.0 | 10.0 | 65.47% (7954) | 5.87 | 1.99 | 0.02 | -0.49 |
| Headaches or Migraines | 1.0 | 10.0 | 37.33% (4535) | 5.38 | 2.03 | 0.13 | -0.5 |
| Memory Changes | 1.0 | 10.0 | 23.03% (2798) | 5.07 | 2.05 | 0.26 | -0.45 |
| Numbness of Tingling | 1.0 | 10.0 | 19.07% (2317) | 5.02 | 2.05 | 0.26 | -0.51 |
| Sleep Disturbances | 1.0 | 10.0 | 50.33% (6114) | 5.6 | 2.01 | 0.06 | -0.47 |
| **Respiratory** |  |  |  |  |  |  |  |
| Coughing | 1.0 | 10.0 | 16.98% (2063) | 4.58 | 2.13 | 0.45 | -0.43 |
| Excessive Mucus or Nasal Stuffiness | 1.0 | 10.0 | 31.68% (3849) | 5.0 | 2.06 | 0.28 | -0.56 |
| Irritated or Sore Throat | 1.0 | 10.0 | 22.35% (2715) | 4.48 | 2.02 | 0.47 | -0.3 |
| Snoring | 1.0 | 10.0 | 15.24% (1851) | 4.59 | 2.0 | 0.35 | -0.32 |
| Wheezing or Chest Tightness | 1.0 | 10.0 | 11.02% (1339) | 4.8 | 1.98 | 0.3 | -0.5 |
