# Supplementary material for: The Development of a Digital Patient-Reported Outcome Measurement for Adults With Chronic Disease (The Parsley Symptom Index): Prospective Cohort Study
Source: JMIR Form Res. 2021 Jun 11;5(6):e29122. doi: 10.2196/29122 (PMC8235288; doi:10.2196/29122)
Supplement: Multimedia Appendix 2 [file formative_v5i6e29122_app1.docx]

Table 1: Sample Descriptives

|  |  | Value |
| --- | --- | --- |
| N |  | 4619 |
| Age: mean (SD) |  | 38.9 (11.3) |
| Biological Sex: n (%) | Female | 3706 (80.2) |
|  | Male | 902 (19.5) |
|  | Other/Prefer not to say ^a^ | 11 (0.2) |
| Location: n (%) | Parsley Center LA | 961 (20.8) |
|  | Parsley Center NYC | 3051 (66.1) |
|  | Parsley Center SF | 607 (13.1) |
| Number of Encounters: n (%) | 1 Clinician Encounter | 1033 (22.4) |
|  | 1 to 2 Clinician Encounters | 2268 (49.1) |
|  | 4 or more Clinician Encounters | 1318 (28.5) |
| Membership Duration: n (%) | 1 year or more | 2647 (57.3) |
|  | Less than 1 year | 1972 (42.7) |
| PSI Completion: n  (%) | Completed | 4329 (93.7) |
|  | Not complete | 290 (6.27) |
| *^a^* Gender and biological sex nomenclature are evolving areas; these responses reflect the descriptors used in the questionnaire. | | |
